# A multi‐faceted construct to guide geriatric dental education: Findings from a scoping review with consultation

**DOI:** 10.1111/ger.12769

**Published:** 2024-06-14

**Authors:** Alicia C. Brandt, Lorelei Lingard, Cecilia S. Dong

**Affiliations:** ^1^ Schulich School of Medicine & Dentistry University of Western Ontario London Ontario Canada; ^2^ Centre for Education Research & Innovation University of Western Ontario London Ontario Canada; ^3^ Pathology and Laboratory Medicine University of Western Ontario London Ontario Canada

**Keywords:** dental care for aged, dental education, dental students, geriatric dentistry, willingness to treat

## Abstract

**Background and objectives:**

Older adults report unmet oral health care needs and barriers in access to care, due in part to provider attitudes and discomfort towards treating older patients. Our study asked: What is known from the literature about the use of undergraduate dentistry programmes to influence dental students' attitudes, perceptions and comfort towards treating geriatric patients? And how can interdisciplinary care facilitate the ability of dentists to work with geriatric patients?

**Materials and methods:**

A scoping review and stakeholder consultation followed established methodological guidelines. Four databases and two grey literature sources were searched. Two researchers independently selected articles using predefined inclusion criteria. Pertinent information was inputted into an iteratively developed extraction table. NVivo 12 was used to organise the extracted data into themes. Key findings were confirmed through stakeholder consultation.

**Results:**

Sixty‐eight articles were included in the scoping review. Five key themes emerged: (1) Curricular targets; (2) Intervention components; (3) Dentist and patient factors; (4) The role of interdisciplinary care; and (5) Post‐graduation insights on knowledge‐seeking patterns. Stakeholder consultations involved 19 participants from Southwestern Ontario and generally confirmed our findings.

**Conclusions:**

Inconsistent reporting of multiple intervention dimensions constrains our ability to strengthen this knowledge. Future interventions and their reporting could be improved by adopting “willingness to treat” as an overarching, multi‐faceted concept which encompasses knowledge on ageing, attitudes towards older patients, perceived competence and empathy. Stakeholder interviews complemented these findings.

## 1 INTRODUCTION

Older adults have increasingly complex health and dental care needs and report unmet oral care needs. This need will continue increasing as the population ages.^1^, ^2^, ^3^ Older adults now have a better understanding of preventive care, and attitudes have shifted towards keeping natural teeth.^2^, ^4^ This presents an opportunity for dental professionals to more frequently assess oral and overall health in order to provide appropriate preventive care for this population, which is critical because oral health complaints may be an indicator of underlying social or systemic health concerns.^5^, ^6^ Chronic disease screenings conducted by dentists have been determined to increase detection and referrals for diabetes, hypertension and hypercholesterolemia and save up to $102.6 million healthcare dollars.^7^ Using an interdisciplinary care model, early identification of health problems by a dental professional could lead to more timely referrals and better comprehensive geriatric care.^7^, ^8^

Older adults experience complex barriers in access to care. Although 70% of older adults are functionally independent, 60%–90% report needing dental care, while only 20% report accessing it.^9^, ^10^ Income closely aligns with care‐seeking patterns; however, even when financial barriers are removed, the volume of patients seeking care does not increase proportionately.^3^ This has been attributed, in part, to underlying negative opinions of dental professionals towards treating older patients: in one survey of Canadian dentists, 80% of responses contained indications of ageism, and two‐thirds of dentists indicated no interest in or willingness to treat geriatric patients.^3^

A culture and attitude change towards positive perceptions and comfort in treating older adults is required at the dental school level.^1^, ^2^, ^11^, ^12^ However, persistent negative attitudes have been identified in dental students towards geriatric patients, and levels of discomfort in treating older patients with complex needs.^2^, ^13^, ^14^ Furthermore, a focused scoping review of peer‐reviewed literature on undergraduate geriatric dental education reported limited recent research on the educational outcomes in geriatric dental curricula, and a lack of both curricular guidelines and standardised language.^15^ Building on this work, we designed a scoping review with a widened search timeline and expanded data sources, in order to provide a more inclusive description of undergraduate geriatric dental education.

## 2 METHODS

### 2.1 Review structure

We followed established scoping review guidelines^16^, ^17^, ^18^ to answer two research questions:

1. What is known about the use of undergraduate dentistry programmes to influence dental students' attitudes, perceptions and comfort towards treating geriatric patients?
2. How can interdisciplinary care facilitate the ability of dentists to work with geriatric patients?

### 2.2 Eligibility criteria

An academic librarian assisted in developing a search strategy and inclusion/exclusion criteria (Table 1). For standardisation across studies, an older adult was defined as anyone over age 65, following Statistics Canada.^19^

**TABLE 1:** Inclusion and exclusion criteria.

Inclusion criteria	Exclusion criteria
Discusses undergraduate dental programmes or supplements	Does not discuss undergraduate education or dental students or discusses these groups but pools data with other groups, where dental student specific data cannot be isolated.
Discusses attitudes, perceptions, comfort or preparedness of dentists or students.	Does not discuss dental student or practising dentist attitudes, perceptions or comfort in treating geriatric patients.
Focus on treating geriatric dental patients with mean age 65 or older	Discusses patients with a mean age below 65 years.
Published in English	Not published in English

### 2.3 Search strategy and information sources

Four electronic databases were searched: Medline (Ovid), Embase (Ovid), Scopus and the Cochrane Library. Grey literature was searched using CADTH Grey Matters, Google and Google Scholar; the first 20 pages of results were screened for eligibility, following published recommendations.^20^ Reference lists of articles included after full‐text review were hand‐searched. A search strategy was iteratively developed using free text and Medical Subject Headings and trialled in Medline. Search terms included “dental students” or “dental education,” “attitudes” or “comfort,” and “geriatric patients.” The search was limited to 1 January 1970 to December 2022, since geriatric dentistry was not identified as a concept until 1970.^2^ Searches were conducted in May 2020 and updated in December 2022. The first three pages of results were reviewed, and the search revised until a comprehensive strategy was developed. This strategy was executed in each database and results were imported into Mendeley reference management software for duplicate removal and screening.

### 2.4 Study selection

Titles and abstracts were reviewed by two authors (ACB and CSD). Articles meeting inclusion criteria were retrieved for an independent full‐text review by both authors (ACB and CSD), who met weekly to ensure calibration and to reach a consensus on articles for which there was disagreement.

### 2.5 Data collection and analysis

All selected articles were read completely, and the relevant information entered into an iteratively developed extraction table (Table 2). Both authors trialled extracting one article and revised the extraction categories: the final extraction categories reflected their consensus. Extracted data were qualitatively analysed following line‐by‐line coding^21^, ^22^ using NVivo 12. Generated nodes were hand sorted and organised into themes using an inductive approach.^21^ This review protocol was registered ad hoc with Open Science Framework.

**TABLE 2:** Sample extraction table categories.

Article ID
Author
Year
Title
Purpose
Country of origin
Methods
Geriatric patient category (eg, community, long‐term care and other)
Programme/intervention description
Interdisciplinary component? (yes/no)
Key findings
Notes

### 2.6 Stakeholder consultations

A consultation process was conducted after data analysis to gather perspectives from dental students, dentists and dental educators on our results.^17^ This portion of the study was reviewed and received ethics clearance from the University of Western Ontario's Non‐Medical Research Ethics Board (2021‐119 213‐55594**).** We presented a two‐page lay summary of findings to participants from Southwestern Ontario who had been recruited purposively through networks within the University of Western Ontario Schulich School of Medicine and Dentistry and via snowball sampling for individual interviews with two aims: (1) To review a summary of our results; and (2) To engage in a discussion of implementing geriatric dentistry curriculum changes provincially in Ontario, Canada. Interviews were conducted over Zoom, audio‐recorded and transcribed verbatim. The section of the interview pertaining to our first aim was analysed separately line‐by‐line into codes using NVivo 12.^22^ Codes were then printed and hand sorted following Braun and Clarke's^21^ thematic analysis to derive themes inductively. We include the interview findings from our first aim in this report; the results of the implementation discussion feature in a separate publication.^23^

## 3 RESULTS

**FIGURE 1:**
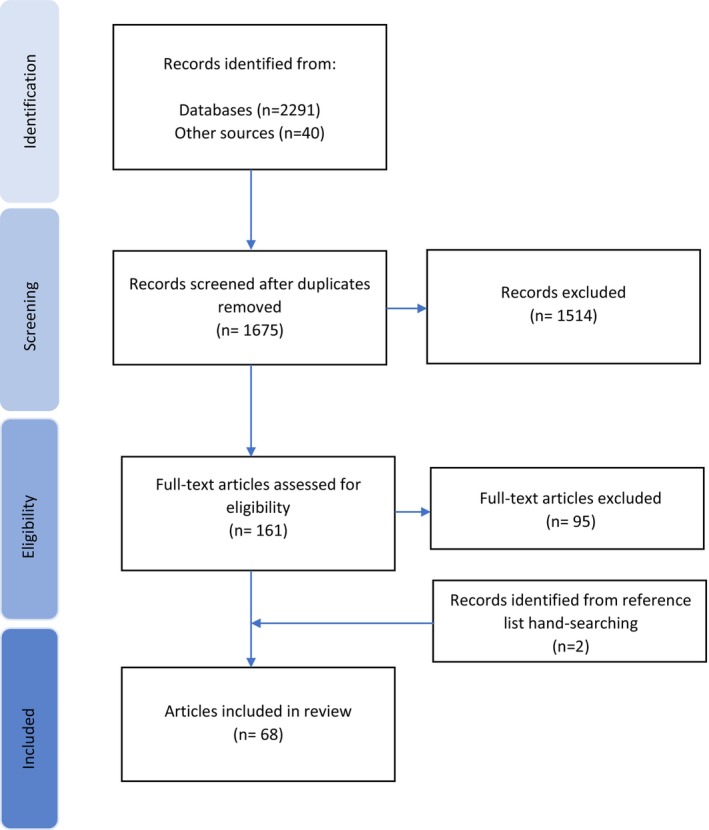
PRISMA flow diagram of considered studies.

A total of 1675 articles underwent title and abstract review. One hundred sixty one full‐text articles were obtained for further review, and 66 articles were included. A reference list search identified two articles for a total of 68 articles included in the review (Figure 1). The majority of articles originated from the United States (40%), but articles also originated from 20 additional countries (Table 3). Over 50% of articles had been published in 2010 or later; the majority included clinical students (71%), and the source of older adult patients was undefined in almost two‐thirds (65%). Most articles did not include interdisciplinary component (90%).

**TABLE 3:** Characteristics of included studies (*n* = 68).

Country of origin	Frequency^a^	%
United States	27	40
India	6	9
Iran	5	7
United Kingdom	4	6
Brazil	3	4
Canada	3	4
Chile	2	3
Japan	2	3
Malaysia	2	3
Greece	2	3
Thailand	1	1
Saudi Arabia	2	3
Argentina	1	1
Australia	1	1
Belgium	1	1
Colombia	1	1
Croatia	1	1
Germany	1	1
Indonesia	1	1
Ireland	1	1
Malta	1	1
Pakistan	1	1
Peru	1	1
Spain	1	1
Switzerland	1	1
Turkey	1	1
Year of publication		
1970–1979	2	3
1980–1989	10	15
1990–1999	6	9
2000–2009	12	18
2010–2019	28	41
2020–2022	10	15
Source of patient population		
Dental clinic	7	10
Long‐term care	12	18
Home care	1	1
Community or public health setting	1	1
Outpatient hospital clinic	1	1
Veterans administration clinic	1	1
Undefined	48	71
Student population		
Preclinical	23	34
Clinical	57	84
Unspecified dental student group	7	10
Interdisciplinary component included		
Yes	7	10
No	61	90

^a^ Some articles included multiple sources for patient populations and multiple student populations.

Five themes were identified through the analysis of key findings in the extraction table: (1) Curricular targets; (2) Intervention components; (3) Dentist and patient factors; (4) The role of interdisciplinary care; (5) Post‐graduation insights and knowledge‐seeking patterns. These themes and their subthemes are described below.

### 3.1 Curricular targets

Geriatric dentistry curricula contain four main curricular targets or subthemes: Knowledge about ageing; Attitudes towards older patients; Perceived competence; and Empathy.

#### 3.1.1 Knowledge about ageing

Twenty‐six papers discussed knowledge about ageing. Dental students demonstrated higher scores in general knowledge, delivering and maintaining care and understanding oral conditions older people.^24^, ^25^, ^26^ They possessed low scores related to psychological issues older adults face, social aspects of care and health behaviours of older people.^24^, ^25^, ^26^, ^27^ Overall, students had low or moderate knowledge scores.^24^ Relative to other student types, dental students underperformed.^28^, ^29^, ^30^ Some studies found lower knowledge scores were associated with fewer geriatric dentistry curriculum components and lower attitude scores.^28^, ^30^ while other studies found knowledge on ageing had minimal or no effect on attitudes towards ageing, or willingness to treat.^26^, ^31^ One study found first‐year dental students, who have a more didactic‐heavy curriculum than senior students, had the highest knowledge scores.^1^ Other studies identified that medical students who had undergone clinical training had the highest knowledge scores.^28^, ^29^

#### 3.1.2 Attitudes towards older patients

Attitudes towards older patients were discussed in 48 articles. Attitudes were often assessed as multi‐faceted, with domains on a continuum. Particular domains scored differently, with some more significantly affected by intervention. For instance, the Personal Acceptability‐Unacceptability subscale on the Ageing Semantic Differential (ASD) commonly had the most positive score;^31^, ^32^ however, it remained the most stable.^33^, ^34^ Patient characteristics also affected attitudes scores. For example, when studying institutionalised patients, scores were lower, particularly on the Instrumental‐Ineffective subscale.^35^, ^36^ Similar patterns occurred when assessing patients of different ages: older geriatric patients tended to receive lower attitude scores than younger ones.^32^ Attitude scores were not affected by profession alone when examining scores in dental and medical students.^29^ While the literature was divided on which interventions may affect attitudes best, it was apparent that attitudes needed to be parsed into domains for valid measurement and that attitude scores were volatile and modifiable with intervention.^1^, ^31^, ^36^, ^37^

#### 3.1.3 Perceived competence

Perceived Competence was explored in 18 articles. Students reported feeling competent in some areas (eg, taking medical and drug histories),^38^ but not others (eg, dealing with underserved populations, like homebound patients).^39^, ^40^ There may a dose effect: Some studies indicated that insufficient clinical exposure reduced perceived competence, while others found that a geriatric curriculum increased comfort in treating older adult patients.^40^, ^41^, ^42^, ^43^ Perceived competence was separated from reported comfort levels in treating different types of patients, but perceived competence appears closely related to comfort in providing different dental services or treating different types of older patients.^40^

#### 3.1.4 Empathy

Empathy and cynicism were introduced in eight articles, often together. Higher empathy scores were linked to positive attitude scores.^37^, ^44^ Empathy was positively and negatively affected by different interventions, and many authors suggested additional research was required to determine how to promote increase empathy.^44^, ^45^, ^46^ Cynicism deteriorated attitude scores and appeared to increase with clinical year.^47^, ^48^ Aside from one article published in 1984, no research examined this area until Waldrop's pivotal 2016 article^37^ which suggested a link between empathy and attitudes. Following this, new research in the area emerged almost yearly.

### 3.2 Intervention components

Most studies measured curricular targets at a single time point. Curricular components included: didactic components, simulation, clinical exposure and extramural rotations. Several articles poorly described their curricular context.

#### 3.2.1 Didactic components

Thirty articles reported on didactic components. Some described and evaluated a specifically designed geriatric dentistry curriculum while others described geriatric dentistry material embedded in other dental course offerings. Many studies showed that didactic training improved knowledge about ageing and reduced biases associated with ageing,^25^, ^26^, ^49^ but the effect of didactic training on attitudes and perceived competence had mixed results.^48^, ^49^, ^50^, ^51^ Almost all studies found didactic training had no effect or a negative effect on attitudes.^26^, ^48^, ^50^ One study found that didactic training had no effect on perceived competence for some students and negatively impacted other students,^52^ while several other studies noted that it can reduce students' perceived competence in treating older patients.^41^, ^51^ Some studies also reported that students did not perceive their didactic training to be adequate and sought information on geriatric dentistry outside school.^53^, ^54^

#### 3.2.2 Simulations

Only two studies discussed simulation. For instance, one used patient actors and simulated case scenarios of ailments commonly faced by older adults^55^ and reported that simulation improved communication skills, treatment planning for complex patients, pain management and management of physical disabilities.^55^

#### 3.2.3 Clinical exposure

Twenty‐four articles described clinical exposures, classified as student experiences within a dental school‐run clinic. Clinical exposures resulted in many positive outcomes, including reported improved communication skills,^56^ better understanding of issues important to older patients,^57^ better diagnosis and treatment planning skills,^56^ reduced misconceptions about older adults^57^ and improved empathy.^37^ With respect to other curricular targets, results were mixed. For instance, clinical experiences were found to have positive, negative and neutral effects on attitudes; effects would also change between positive negative and neutral within a study at different time points based on the duration of exposure.^1^, ^37^, ^41^, ^42^ Other studies reported that students did not feel their clinical exposure was adequate, and there were mixed results on the effect on perceived competency.^52^, ^55^ A dose–response was suggested between clinical exposures and attitudes and perceived competence, where longer exposures are required for positive changes.^31^, ^41^ The quality of the exposure and case variety also appeared important—benefit was seen when students were introduced to older adult patients with good oral health earlier in their training, prior to introduction to more complex cases, and when case portfolios contained both young and old patients.^41^, ^42^, ^58^

#### 3.2.4 Extramural rotations

Nineteen articles discussed extramural rotations, defined as student placements outside of the main school dental clinic. These included long‐term care facilities, hospitals, community mobile units and specialised clinics within the dental school. Rotations improved attitudes towards older patients and comfort in treating complex patients in some cases.^39^, ^50^ However, they could also have a negative effect on enthusiasm and comfort in treating older adult patients once students graduated, and in some cases, attitudes were also negatively affected.^38^, ^39^, ^59^ Other studies found that their extramural rotations did not have an effect on perceived competence or comfort, and attitudes remained unchanged.^20^, ^27^, ^39^, ^48^ The authors suggested that both the quality of the extramural exposure and students' previous experiences play an important role in the overall outcome.^36^, ^57^, ^60^

### 3.3 Dentist and patient factors

Outcomes were also affected by intrinsic provider and patient factors.

#### 3.3.1 Provider factors

Provider factors included their culture, number and quality of social contacts with older persons, implicit biases and gender. In cultures with respect for ageing^32^, ^61^ or a duty to care for older people,^24^, ^62^ providers had more positive attitudes or were more willing to treat older adults,^32^, ^61^, ^63^, ^64^ Social contact with older adults may improve curricular target scores; however, it remains unclear which type of contact elicits positive effects. Studies reported mixed findings, with some concluding clinical contact was beneficial and non‐work‐related contact was not,^13^, ^65^ while others found any contact with older adults could improve willingness to treat.^24^, ^63^ Ultimately, the quality, context and dosage of social contacts with older adults appear to be important variables.^37^, ^65^ The relationship between gender and treatment behaviours remains unclear, with studies disagreeing on the influence of gender on outcomes.^24^, ^29^, ^30^, ^31^, ^39^, ^46^, ^49^, ^50^, ^54^, ^62^ Age was also considered in several studies but was not shown to affect outcomes.^13^, ^29^, ^31^, ^37^, ^46^

#### 3.3.2 Patient factors

Individual patient factors which affected providers' opinions on treatment included their age,^29^, ^33^, ^66^ oral health condition,^32^, ^34^, ^67^ finances,^62^ their level of appreciation for receiving treatment and their compliance with treatment and oral hygiene instruction.^62^

### 3.4 The role of interdisciplinary care

Seven articles included interprofessional education (IPE) and collaboration; almost all suggested it could benefit dentistry. While some studies noted IPE may not significantly impact attitudes or willingness to treat older patients work with other disciplines,^60^ and may hinder effective communication,^60^ many studies found positive responses towards interdisciplinary education and collaboration after students were exposed to it.^1^, ^36^, ^43^ In particular, students requested access to additional interdisciplinary training resources after exposure to more complex older patients and reported feeling significantly more prepared to treat older patients.^36^, ^43^ The authors suggested students should be at the same level of training for interdisciplinary collaboration to be effective^60^ and that interdisciplinary care can facilitate treatment planning and patient management for medically complex patients.^44^, ^51^, ^68^

### 3.5 Post‐graduation insights on knowledge‐seeking patterns

Seven studies examined treatment behaviours and knowledge‐seeking patterns of dentists after graduation, with mixed results. Some found new dentists were more enthusiastic and willing to treat medically complex and older patients,^68^ whereas others found newer dentists possessed more stereotypes about older people and that there was a negative change in willingness to treat frail older people.^45^ Another study reported that there was no difference in graduation year^39^ or the number of years since graduation^40^ on willingness to treat older patients.

On the job training was reported as one of the most popular training methods; however, it was infrequent.^38^ Rather, journal articles and continuing education courses on geriatric dentistry were the most cited sources of information used.^38^

### 3.6 Stakeholder consultations

Ten clinical dental students and nine clinical dental faculty members from one dental school in Ontario were consulted. Male stakeholders comprised 61% of the sample while females comprised 39%. Stakeholders represented a variety of ethnic backgrounds, including Caucasian (8), South‐ (2), East‐ (5) and West‐ (2) Asian and Latin American (2) Most stakeholders confirmed the data aligned with their experiences in dentistry. Students confirmed our findings and described their resonance with their experience: “I was nodding my head in agreement …. you can kind of have a willingness to treat, but you just feel like you don't have the competence … I feel for these people, I want to treat them, they're my patients, but I do feel like maybe we weren't fully‐ we aren't fully prepared” [Dental student, female, ST_02]. Faculty also confirmed the resonance of our findings, commenting that “it's a very good summary actually. Yeah, it's not surprising at all. I've been there. I was a student avoiding geriatrics.” [Faculty, male, FD_10]. Faculty also confirmed particular findings, such as the influence of social/cultural factors:

I personally come from a society where the elder‐ like grandpa and grandma are very well‐respected and we do everything for them. We care for them, we have them in our houses, so that's why when you mentioned about you know cultural and social like aspect of it, I was surprised and was like “oh yes, it makes sense. [Faculty, female, FD_02]

Some faculty stakeholders expressed surprise about particular findings, such as:

Attitude toward older adults, because like I, I haven't experienced this at all from where I came from. Treating geriatric patients at my previous dental experience was, it was mandatory, and it was for free… so that's why I haven't experienced any attitude problem. [Faculty, female, FD_04]

Such surprises, however, tended to be accompanied by acceptance of the finding:

I like the way that you explained. I just wasn't expecting that by being exposed more to having classes regarding these old patients would ‐ had a bad outcome. [Faculty, female, FD_09]

While both students and faculty reflected on the findings in relation to their experience with geriatric dentistry, students generally did not engage further, whereas faculty provided additional explanations of these findings. These included training on evidence‐based outcomes and how these outcomes change for older adult patients, disabilities more commonly faced by older patients, and dentist experience level:

I think that the factor that I don't see here is the dentist experience… I believe that because it's‐ like some complex procedures, it's frustrating for both patients and for the dentist as well having to… repeat the work… I will add the complexity of the case, and the patient factor. [Faculty, female, FD_04]

Interestingly, disability was the only factor mentioned by both groups as a possible modifier of willingness to treat that was not identified in our results. Faculty were explicit that “A lot of older adults will have one or more disabilities and you haven't included that word.” [*Faculty, male, FD_01*], whereas students used disability as an example of challenges that make them uncomfortable:

We aren't fully prepared to treat much older adults, especially older adults who have special health care needs who come in wheelchairs, or you know have disabilities or anything like that. [Dental student, female, ST_02]

While interdisciplinary care was presented and its meaning explained, no participants commented on this.

## 4 DISCUSSION

This scoping review has provided an inclusive overview of the geriatric dental education curricula globally. Although four key curricular targets were identified for changing outcomes in this field, there is significant variability in the impact of interventions on each of these targets. The amount, timing and quality of the exposure appears to impact outcomes; however, the literature currently lacks high‐quality longitudinal data to explore these relationships. It remains unclear which interventions are effective in positively impacting students and which are not. Of paramount importance is the finding that some interventions can have negative effects on curricular targets.

**FIGURE 2:**
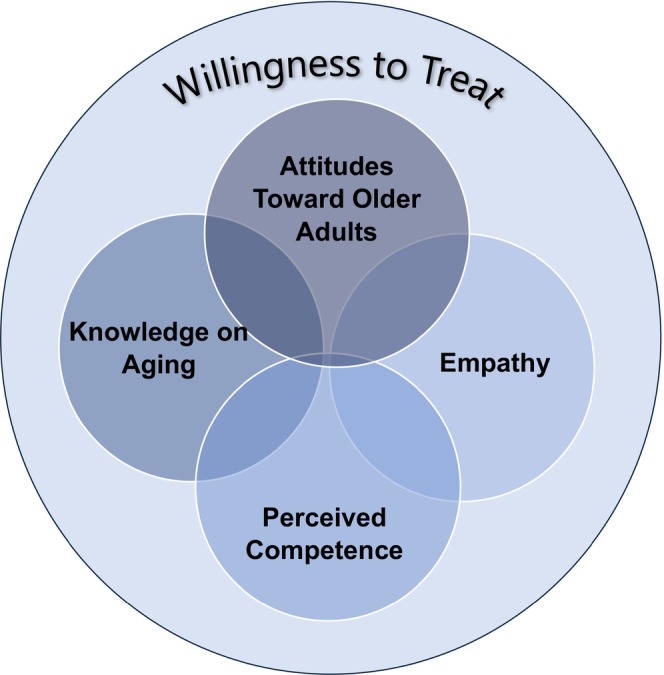
Conceptualisation of willingness to treat. Willingness to treat viewed as a broad concept encompassing attitudes, knowledge, competence and empathy, all of which are interrelated and affect one another. Facets are shaded darker to demonstrate heavier coverage in the current literature and lighter to demonstrate lighter coverage.

The isolated study of individual curricular targets, despite their apparent interrelated nature, may be a root cause of the variability in results identified. Thus, for longitudinal success in improving dentist treatment behaviours a multi‐faceted intervention will be required, encompassing multiple curricular targets. Because each curricular target is being used to modify one common outcome of interest—whether or not a provider would treat a geriatric dental patient—we propose that together, these targets comprise a larger, multi‐faceted construct: “willingness to treat.” This appears to be the true variable of interest in geriatric dental education research. We define willingness to treat as the disposition that shapes a provider's ultimate behaviour of treating a geriatric dental patient, and we conceptualise it as a complex construct affected by several different factors, most prominent of which in the reviewed literature are knowledge of ageing, attitudes towards older patients perceived competence in treating older people and empathy towards older adults. This concept has been previously identified as a singular term but was neither defined nor adopted as an intervention target.^39^, ^40^ Emerging research has begun to adopt the use of willingness to treat as a study concept in the context of increased access to care and provider attitudes towards treating older adults; however, the authors call for additional interventions targeted at improving provider willingness to treat.^69^ We propose that *willingness to treat* be adopted as the common, multi‐faceted, modifier in geriatric dentistry curricular efforts (Figure 2). This would encourage the development of multi‐faceted interventions and support standardisation in reporting, so that studies could be compared in terms of which facets of the construct are being addressed. This would support more robust synthesis of interventions going forward.

IPE has been studied extensively in the health literature;^70^, ^71^, ^72^ however, its incorporation into dentistry is limited. Interventions which do incorporate allied health professionals show successes in several domains, but outcomes are not reported consistently across studies.^73^ One curriculum pairing dental with social work students improved dental students' abilities to appropriately navigate difficult patient management scenarios such as functional or cognitive decline. Students also reported greater awareness of the roles of allied health professionals.^24^ Barriers to IPE include inconsistency in defining learning outcomes, and difficulty accessing other learner groups in siloed dental settings. One review found that dentists specifically had lower scores related to interprofessional collaborative practice after IPE interventions.^73^ A 2023 editorial by three geriatric dental education scholars suggested that a positive first step in dental education would be to incorporate education about various professional roles required for comprehensive management of older patients.^74^ Interestingly, although IPE appears to be an emerging recognised need in dentistry, our stakeholders had little to say about it. Given their other responses were rich in detail, this gap in the dental stakeholder interviews may suggest that they are unacquainted with the idea of IPE. Given that ideal care for medically complex geriatric patients is best provided within an interdisciplinary care model,^71^ demographic trends towards older, complex patients retaining their natural teeth longer and that a recent scoping review concluded dental students have the potential to develop positive attitudes through training,^75^ we strongly suggest that the delivery of future geriatric dental care would benefit from integrating IPE into undergraduate dental education to increase adoption of interdisciplinary collaboration after graduation.

One limitation of this study was that its broad scope required an analytical overview rather than more granular analysis of specifics such as methods. Previous work has provided this granular analysis of peer‐reviewed literature post‐2009,^15^ and our overview is intended to broaden and build on its findings. We echo earlier findings that educational outcomes are poorly reported in geriatric dental curricula and that standardised language and definitions are lacking to inform geriatric dentistry curricular development. Our primary novel contribution addresses both of these problems by defining a comprehensive, multi‐faceted construct—willingness to treat—to support future standardisation and robust outcomes reporting.

## 5 CONCLUSION

Lack of standardisation in reporting makes synthesis of geriatric dentistry curricula interventions difficult. Our scoping review suggests four factors that affect a dental student's choice to treat an older patient: knowledge about ageing, attitudes, perceived competence and empathy. Currently, these factors are treated separately; we propose integrating them into the multi‐dimensional construct of “willingness to treat” as the outcome of interest in geriatric dentistry educational interventions. Standardised reporting using this construct would allow explorations of possible dose, case complexity and implementation timing effects on the dimensions of willingness to treat. Finally, while interdisciplinary education appears promising to support development of willingness to treat, its wider implementation within geriatric dentistry curricula is necessary to understand its impact.

## AUTHOR CONTRIBUTIONS

ACB, LL and CSD made substantial contributions to the conception and design of the work. ACB and CSB conducted data acquisition and analysis and ACB drafted the work for review. LL and CSB provided substantial contributions to the critical review and revisions of the work. All authors are accountable for this work.

## FUNDING INFORMATION

Schulich Dentistry Research Studentship, Schulich School of Medicine and Dentistry. This study was reviewed and received ethics clearance from the University of Western Ontario's Non‐Medical Research Ethics Board (2021–119 213‐55 594**).**

## CONFLICT OF INTEREST STATEMENT

The authors declare no conflict of interest.

## Data Availability

Data are available from the corresponding author upon request.

## References

[1] Waldrop DP , Fabiano JA , Nochajski TH , Zittel‐Palamara K , Davis EL , Goldberg LJ . More than a set of teeth: assessing and enhancing dental students' perceptions of older adults. Gerontol Geriatr Educ. 2006;27(1):37‐56. doi:10.1300/J021v27n01_03 16873208

[2] Ettinger RL . A 30‐year review of a geriatric dentistry teaching programme. Gerodontology. 2012;29(2):e1252‐e1260. doi:10.1111/j.1741-2358.2011.00471.x 22612843

[3] Yao CS , MacEntee MI . Inequity in oral health care for elderly Canadians: part 2. Causes and ethical considerations. J Can Dent Assoc. 2014;80:e10. 24598327

[4] Ettinger RL . The new elderly: what can the dental profession expect? Spec Care Dentist. 1982;2(2):62‐69. doi:10.1111/j.1754-4505.1982.tb01282.x 6953609

[5] Avlund K , Holm‐Pedersen P , Schroll M . Functional ability and oral health among older people: a longitudinal study from age 75 to 80. J Am Ger Soc. 2001;49:954‐962. 10.1046/j.1532-5415.2001.49187.x 11527488

[6] Levkoff S , Berkman B , Balsam A , Minaker K . Health promotion disease prevention: new directions for geriatric education. Educ Gerontol. 1996;22(1):93‐104.

[7] Nasseh K , Greenberg B , Vujicic M , Glick M . The effect of chairside chronic disease screenings by oral health professionals on health care costs. Am J Public Health. 2014;104(4):744‐750. doi:10.2105/AJPH.2013.301644 24524531 PMC4025684

[8] Branch‐Mays GL , Pittenger AL , Williamson K , Milone A , Hein E , Thierer T . A interprofessional education and collaborative practice model for dentistry and pharmacy. J Dent Educ. 2017;81(12):1413‐1420. doi:10.21815/JDE.017.101 29196328

[9] Ettinger RL . Oral health and the aging population. J Am Dent Assoc. 2007;138(1):S5‐S6. doi:10.14219/jada.archive.2007.0357 17761839

[10] Marvin MF . Access to care for seniors – dental concerns. J Can Dent Assoc. 2001;67(9):504‐506. 11597341

[11] Kress GC , Vidmar GC . Critical skills assessment for the treatment of geriatric patients. Spec Care Dentist. 1985;5(3):127‐129. doi:10.1111/j.1754-4505.1985.tb00543.x 3859026

[12] Yellowitz J , Saunders MJ . The need for geriatric dental education. Dent Clin N Am. 1989;33(1):11‐18. 2642818

[13] Wood GJ , Mulligan R . Cross‐sectional comparison of dental students' knowledge and attitudes before geriatric training: 1984–1999. J Dent Ed. 2000;64(11):763‐771. doi:10.1002/j.0022-0337.2000.64.11.tb03380.x 11191878

[14] Hatami B , Ebn Ahmady A , Khoshnevisan MH , Lando HA . Dental students' perceived barriers in geriatric dental care active involvement. Oral Health Dent Manag. 2014;13(3):675‐679. 25284535

[15] Nilsson A , Young L , Glass B , Lee A . Gerodontology in the dental school curriculum: a scoping review. Gerodontology. 2021;38(4):325‐337. doi:10.1111/ger.12555 33977554

[16] Arksey H , O'Malley L . Scoping studies: towards a methodological framework. Int J Soc Res Methodol Theory Pract. 2005;8(1):19‐32. doi:10.1080/1364557032000119616

[17] Levac D , Colquhoun H , O‐Brien KK . Scoping studies: advancing the methodology. Implement Sci. 2010;5:69. doi:10.1186/1748-5908-5-69 20854677 PMC2954944

[18] Peters MDJ , Marnie C , Tricco AC , et al. Updated methodological guidance for the conduct of scoping reviews. JBI Evid Implement. 2021;19(1):3‐10. 33570328 10.1097/XEB.0000000000000277

[19] Statistics Canada . Seniors. 2018. Accessed July 26, 2020. https://www150.statcan.gc.ca/n1/pub/11‐402‐x/2011000/chap/seniors‐aines/seniors‐aines‐eng.htm

[20] Haddaway NR , Collins AM , Coughlin D , Kirk S . The role of Google scholar in evidence reviews and its applicability to grey literature searching. PLoS One. 2015;10(9):e0138237. doi:10.1371/journal.pone.0138237 26379270 PMC4574933

[21] Braun V , Clarke V . Using thematic analysis in psychology. Qual Res Psychol. 2008;3(2):77‐101. doi:10.1191/1478088706qp063oa

[22] Lofland J , Snow DA , Anderson L , Lofland LH . Analyzing Social Settings: A Guide to Qualitative Observation and Analysis. 4th ed. Wadsworth Publishing; 2006.

[23] Brandt AC , Abbas A , Dong CS . Exploring the acceptance of geriatric dentistry programming for undergraduate dental students through stakeholder interviews. J Dent Educ. 2024;88(5):573‐586. doi:10.1002/jdd.13461 38321860

[24] Hatami B , Ebn Ahmady A , Khoshnevisan MH , Lando HA . Senior dental student's attitudes toward older adults and knowledge of geriatric dental care in the Islamic Republic of Iran. East Mediterr Health J. 2014;9(19 suppl 3):S172‐S177. 24995742

[25] Fabiano JA , Waldrop DP , Nochajski TH , Davis EL , Goldberg LJ . Understanding dental students' knowledge and perceptions of older people: toward a new model of geriatric dental education. J Dent Educ. 2005;69(4):419‐433. 15800255

[26] Anehosur GV , Nadiger RK . Evaluation of understanding levels of Indian dental students' knowledge and perceptions regarding older adults. Gerodontology. 2012;29(2):e1215‐e1221. doi:10.1111/j.1741-2358.2010.00416.x 20825498

[27] Husna AA , Robaiyah K , Tanti IR . Dental Students' knowledge and perception of elderly in relation to geriatric dentistry training. Med Health. 2009;4:76‐83. Accessed June 16, 2021. https://api.semanticscholar.org/CorpusID:263563742

[28] Friedman PK , Brecknock S . A comparison of dental and dental hygiene students' performance on "facts of aging" quiz. J Mass Dent Soc. 2003;52(1):36‐39. 12825567

[29] Holtzman JM , Beck JD , Ettinger RL . Cognitive knowledge and attitudes toward the aged of dental and medical students. Educ Gerontol. 1981;6(2–3):195‐207. doi:10.1080/0380127810060210

[30] Steele LP . Dental students' attitudes and knowledge about elderly people. Gerodontics. 1987;3:61‐64. 3472982

[31] Nochajski TH , Davis EL , Waldrop DP , Fabiano JA , Goldberg LJ . Factors that influence dental students' attitudes about older adults. J Dent Educ. 2009;73(1):95‐104. doi:10.1002/j.0022-0337.2009.73.1.tb04642.x 19126770

[32] Ettinger RL , Beck JD , Lekfuangfu S , Luangjamekorn V . Attitudes towards the elderly in a group of Thai dental students. Odontostomatol Trop. 1986;9(1):7‐14. 3460052

[33] Sheiham A , Ettinger RL , Beck JD . English dental students' attitudes towards the aged. Gerodontics. 1986;2(4):142‐146. 3462079

[34] Beck JD , Ettinger RL , Glenn RE , Paule CL , Holtzman JM . Oral health status: impact on dental student attitudes toward the aged. Gerontologist. 1979;19(6):580‐584. doi:10.1093/geront/19.6.580 527845

[35] De Visschere L , Van Der Putten GJ , de Baat C , Schols J , Vanobbergen J . The impact of undergraduate geriatric dental education on the attitudes of recently graduated dentists towards institutionalised elderly people. Eur J Dent Educ. 2009;13(3):154‐161. doi:10.1111/j.1600-0579.2008.00555.x 19630934

[36] Brondani M , Pattanaporn K . Dental students' reflections about long‐term care experiences through an existing model of oral health. Gerodontology. 2017;34(3):326‐333. doi:10.1111/ger.12269 28393387

[37] Waldrop DP , Nochajski T , Davis EL , Fabiano J , Goldberg L . Empathy in dentistry: how attitudes and interaction with older adults make a difference. Gerontol Geriatr Educ. 2016;37(4):359‐380. doi:10.1080/02701960.2014.993065 25495912

[38] Ettinger RL , McLeran H , Jakobsen J . Effect of a geriatric educational experience on graduates activities and attitudes. J Dent Educ. 1990;54(5):273‐278. 2335659

[39] Kuthy RA , McQuistan MR , Heller KE , Riniker‐Pins KJ , Qian F . Dental students' perceived comfort and future willingness to treat underserved populations: surveys prior to and immediately after extramural experiences. Spec Care Dentist. 2010;30(6):242‐249. doi:10.1111/j.1754-4505.2010.00161.x 21044104 PMC3003618

[40] McQuistan MR , Kuthy RA , Heller KE , Qian F , Riniker KJ . Dentists' comfort in treating underserved populations after participating in community‐based clinical experiences as a student. J Dent Educ. 2008;72(4):422‐430. 18381848

[41] Cunningham MA , Beck JD , Ettinger RL . Dental students' self‐assessed competence in treating geriatric patients. Spec Care Dent. 1984;4(3):113‐118. doi:10.1111/j.1754-4505.1984.tb00169.x 6589800

[42] Ettinger RL , Beck JD , Kerber P , Scandrett FR . Dental students' confidence in prosthodontics and attitudes toward the elderly. J Dent Educ. 1982;46(9):541‐547. 7050199

[43] Wighton H , Derman SHM , Wicht MJ , et al. Impact of an interdisciplinary curriculum for dental students (GeriDent‐Cologne) on attitudes and awareness towards older people and geriatric conditions. Eur J Dent Educ. 2022;26(3):586‐598. doi:10.1111/eje.12735 34882935

[44] Raval HJ , Mahajan N , Sethuraman R . Comparative evaluation of attitude of teaching faculties and interns of a health sciences university toward geriatric people using Kogan's attitudes toward old people scale: a cross‐sectional study. SRM J Dent Sci. 2018;9(3):103‐107. doi:10.4103/srmjrds.srmjrds_6_18

[45] Major N , McQuistan MR , Qian F . Changes in dental Students' attitudes about treating underserved populations: a longitudinal study. J Dent Educ. 2016;80(5):517‐525. 27139202

[46] Kossioni AE , Ioannidou K , Kalyva D , et al. Translation and validation of the Greek version of an ageism scale for dental students (ASDS _Gr). Gerodontology. 2019;36(3):251‐257. doi:10.1111/ger.12403 30957278

[47] Devlin H , Mellor AC , Worthington HV . Attitudes of dental students towards elderly people. J Dent. 1994;22(1):45‐48. doi:10.1016/0300-5712(94)90144-9 8157811

[48] Eyison J , Mann J , Holtzman JM , Mersel A . A comparative study of the attitude of dental students towards the elderly. Eur J Prosthodont Restor Dent. 1992;1(2):87‐90. 1306741

[49] Sargeran K , Mohebbi SZ , Chinipardaz Z , Gurani Y . Senior dental students' training on oral health care for geriatric patients: an interventional study. J Contemp Med Sci. 2017;3(12):326‐330.

[50] Nitschke I , Clarenbach‐Tran T , Schlegel D , Reiber T , Sobotta BAJ . Attitudes of German undergraduate dental students towards the aged. Gerodontology. 2013;32(1):3‐12. doi:10.1111/ger.12043 23516991

[51] Bittencourt CCD , de Cunha FL , Silva ASF , Zanin L , Florio FM . Impact of the inclusion of geriatric dentistry in the curriculum of a Brazilian dental faculty. Bioscience. 2016;32(4):1118‐1127.

[52] Kiyak HA , Brudvik J . Dental students' self‐assessed competence in geriatric dentistry. J Dent Educ. 1992;56(11):728‐734. 1430528

[53] Purohit A , Purohit BM , Tewari S , Naaz ST , Zainab U . Awareness of geriatric dental care among Indian dental students in central Indian university. ACTA Sci Dent Sci. 2018;2(11):32‐33.

[54] de Lima Saintrain MV , de Souza EH , de França Caldas Júnior A . Geriatric dentistry in Brazilian Universities. Gerodontology. 2006;23(4):231‐236. doi:10.1111/j.1741-2358.2006.00128.x 17105505

[55] Patel SA , Halpin RM , Keosayian DL , et al. Impact of simulated patients on students' self‐assessment of competency in practice of geriatric dentistry. J Dent Educ. 2020;84(8):908‐916. doi:10.1002/jdd.12176 32394449

[56] Patil PG , Ueda T , Sakurai K . Influence of early clinical exposure for undergraduate students on self‐perception of different aspects of geriatric dental care: pilot study between two colleges from Japan and India. J Indian Prosthodont Soc. 2016;16(3):288‐293. doi:10.4103/0972-4052.186402 27621550 PMC5000560

[57] MacEntee MI , Pruksapong M , Wyatt CC . Insights from students following an educational rotation through dental geriatrics. J Dent Educ. 2005;69(12):1368‐1376. 16352773

[58] León S , Correa‐Beltrán G , Giacaman RA . Negative ageing stereotypes in students and faculty members from three health science schools. Gerodontology. 2015;32(2):141‐148. doi:10.1111/ger.12065 23822151

[59] Attard N , Schembri A , Caruana C , Agius AM , Gainza‐Cirauqui ML . Undergraduate students' evaluation and reflections on a gerodontology programme. Eur J Dent Educ. 2018;22(3):e624‐e633. doi:10.1111/eje.12367 29808601

[60] Reilly JM , Aranda MP , Segal‐Gidan F , et al. Assessment of student interprofessional education (IPE) training for team‐based geriatric home care: does IPE training change students' knowledge and attitudes? Home Health Care Serv Q. 2014;33(4):177‐193. doi:10.1080/01621424.2014.968502 25256717

[61] Gupta A , Venkatraman S , Kamarthi N , Goel S , Goel S , Keswani T . Assessment of the attitude of undergraduate dental students toward the geriatric population. Trop J Med Res. 2014;17(2):104.

[62] Devaki T , Mallikapuram KP , Simha BV , Chandu VC , Pavani NPM , Srinivas R . Attitudes and perceived barriers in geriatric dental care among undergraduate dental students in capital region of Andhra Pradesh. J Indian Assoc Public Health Dent. 2020;18(2):134‐138.

[63] Sadaf A , Yazdanie N . Attitude of dental students towards elderly. Pakistan Oral Dental J. 2012;32(1):176‐179.

[64] Tabari ZA , Ghaedi FB , Hamissi JH , Eskandari S . Assessment and attitude of university students about elderly: preliminary study. J Med Life. 2015;8(2):28‐31. PMC5327715 28255393

[65] Poorsattar Bejeh Mir A , Poorsattar Bejeh Mir M . Dentistry students ageing anxiety levels in northern Iran. Gerodontology. 2014;31(4):260‐264. doi:10.1111/ger.12030 23240870

[66] Ettinger RL , Beck JD , Barnard PD , Klineberg IJ . Attitudes of Australian dental students towards the elderly. Aust J Ageing. 1984;3(4):13‐17.

[67] Pyle MA , Jasinevicius TR , Sheehan R . Dental student perceptions of the elderly: measuring negative perceptions with projective tests. Spec Care Dentist. 1999;19(1):40‐46. doi:10.1111/j.1754-4505.1999.tb01367.x 10483460

[68] Fiske J , Gelbier S , Watson RM . Barriers to dental care in an elderly population resident in an inner city area. J Dent. 1990;18(5):236‐242. doi:10.1016/0300-5712(90)90020-f 2074295

[69] Chuang JCP , Pradhan A , Walsh LJ , Lopez Silva CP . Singapore dentists' attitudes toward dental care provision for older adults with disabilities. Gerodontology. 2024;41(1):59‐67. doi:10.1111/ger.12685 36924433

[70] Romanow RJ . Building on values: the future of health care in Canada. Ottawa (ON): Canada, Commission on the Future of Health Care in Canada; 2002. Accessed October 26, 2023. http://publications.gc.ca/collections/Collection/CP32‐85‐2002E.pdf

[71] World Health Organization (WHO) . Framework for action on interprofessional education & collaborative practice. WHO; Geneva, Switzerland. WHO/HRH/HPN/10.3 2010.

[72] Green BN , Johnson CD . Interprofessional collaboration in research, education, and clinical practice: working together for a better future. J Chiropr Educ. 2015;29(1):1‐10. doi:10.7899/JCE-14-36 25594446 PMC4360764

[73] Kossioni AE , Marchini L , Childs C . Dental participation in geriatric interprofessional education courses: a systematic review. Eur J Dent Educ. 2018;22(3):e530‐e541. doi:10.1111/eje.12348 29603840

[74] Johnsen DC , Marchini L , Ettinger RL . How can dental research deliver better outcomes to frail older adults? Essay on considerations. Spec Care Dentist. 2023;43(4):375‐379. doi:10.1111/scd.12802 36715103

[75] Bulgarelli AF , Santos CM , Tôrres LHN , et al. What influences dental students' attitudes regarding the treatment of older adults? A scoping review. J Dent Educ. 2023;87(6):813‐824. doi:10.1002/jdd.13193 36928643

